# A 14-year-old male patient with diagnosis of Prader–Willi syndrome in Ethiopia: a case report

**DOI:** 10.1186/s13256-023-04282-5

**Published:** 2023-12-25

**Authors:** Kibret Enyew Belay, Beza Leulseged Ayalew, Melaku Taye Amogne, Theodros Aberra Alemneh, Tedla Kebede Geletew

**Affiliations:** 1 Department of Internal Medicine, Endocrinology and Metabolism Unit, Bahirdar University, Bahirdar, Ethiopia; 2 Department of Internal Medicine, Endocrinology and Metabolism Unit, Addis Ababa University, Addis Ababa, Ethiopia

**Keywords:** Prader–Willi, Ethiopia, Obesity, Case report, Hypogonadism

## Abstract

**Background:**

Prader–Willi syndrome is a complex multisystem disorder due to the absent expression of paternally active genes in the Prader–Willi syndrome-critical region on chromosome 15 (15q11.2-q13). The main clinical features are hyperphagia (which frequently results in early-onset obesity), hypogonadism, developmental delays, typical behaviors (such as obsessive–compulsive tendencies, tantrums, perseveration, insistence on sameness, and rigidity), and distinctive facial features. In infants, the most prominent findings are hypotonia and feeding difficulties.

**Case presentation:**

This paper highlights a case of a 14 year old male patient of an Ethiopian ethnicity with diagnosis of Prader–Willi syndrome, which is first report in Ethiopia. He presented with progressive excessive weight gain, insatiable appetite, clinical and laboratory features of hypogonadism, ophthalmological refractory error, and facial features of Prader–Willi syndrome, which was further confirmed by genetic analysis. He is currently on lifestyle intervention, testosterone replacement, and treatment for vitamin D deficiency.

**Conclusion:**

Prader–Willi syndrome should be considered in a child who presents with progressive weight gain and other typical clinical features such as cognitive impairment, excessive insatiable eating, or hypothalamic hypogonadism. Early lifestyle intervention may help to reduce excessive weight gain. To our knowledge, this is the first case reported in Ethiopia.

## Introduction

Prader–Willi syndrome (PWS) is a rare human imprinting disorder resulting from genomic alterations that inactivate imprinted, paternally expressed genes in the human chromosome region 15q11–q13 [1]. The genetic mutation results in altered hypothalamus development and function, which impairs satiety, numerous hormonal deficits, changes in sleep microstructure, irregularities in breathing during sleep, and other comorbidities [2]. There are three main classes of chromosomal abnormalities that lead to PWS: deletion on 15q11–q13 (65–75%), maternal uniparental disomy of chromosome 15 (20–30%), or a defect in the imprinting center on 15q11–q13 (2%), although gene mutation (< 0.1%) and balanced translocation (0.1%) can also be found [3]. Recent epidemiological study estimates an incidence of 1 in 25,000 births and a population prevalence of 1 in 50,000 [4]. The birth prevalence for individuals with a molecular diagnosis of Prader–Willi syndrome was estimated to be 1:15,830 from the Victorian Prader–Willi Syndrome register [5].

The vast majority of cases are sporadic. The probability of recurrence in subsequent pregnancies with the same parents is, for the majority of patients, less than 1%; however, the risk is increased in extremely uncommon patients with genetic deletion of the imprinting control center or parental chromosomal rearrangement [6].

Common clinical manifestations include hypotonia, a poor suck, or failure to thrive during infancy [7]. During childhood, they will present with hyperphagia and morbid obesity and will typically have a characteristic facial appearance, short stature, and small hands or feet due to growth and other hormone deficiencies [7]. They will have hypogonadism or hypogenitalism, delayed or incomplete secondary sexual characteristics, weight gain worsening, and behavioral problems in late childhood and adolescence [6].

PWS DNA testing is required if there are characteristic symptoms of the condition such as cognitive impairment, excessive eating, central obesity, and hypothalamic hypogonadism [1]. DNA methylation analysis distinguishes PWS from Angelman syndrome (AS) in deletion cases and aids in the diagnosis of PWS in all three molecular groups. A methylation analysis consistent with PWS is sufficient for clinical diagnosis [8].

## Case presentation

We are reporting a 14 year old male patient of an Ethiopian ethnicity who had been transferred from private clinic 3 months ago with a compliant of progressively increased weight gain since child hood. At birth, he weighed 5 kg, and at 6 weeks old that weight had gradually increased to 8 kg. After his 4th year of age, the weight gain progression became worse. He had increased appetite to food since childhood and favored eating sugar-containing foods. He had a history of acting aggressively, which is followed by irrational rage and emotional outbursts when the size of a meal is reduced or restricted.

His family visited nearby primary health centers during his early childhood and advised for lifestyle management but was unsuccessful. Possible genetic tests were not advised during their initial visits. He is currently in grade 7; he once ranked in the top ten percent of his class, but currently he has been skipping lessons due to illness, going to school occasionally, and doing less well. There was no family history of obesity, diabetes mellitus, hypertension, or other chronic illnesses, except for his overweight mother, who had a body mass index (BMI) of 29kg/m^2^.

His vital signs were all in normal range. He had a weight of 160 kg and a height of 157 cm, making his BMI 65 kg/m^2^, which is class III obesity. He had narrow nasal bridge and thin vermilion of the upper lip with down-turned corners of the mouth. There were dry, dark patches on the skin of the neck and armpit, and there was no hair growth at the armpit or pubic area. He had no secondary sexual characteristics (Tanner stage 1). He had a micro penis and poorly rugated scrotal skin with a small testicle of 1–2 ml size by orchidometer measurement.

Basic laboratory tests, including complete blood count, renal function test, liver enzymes, serum bilirubin, serum albumin, and serum electrolytes, were all normal. Fasting hormonal and lipid profiles are summarized in Table 1. Ophthalmological evaluation showed refractive error corrected with eyeglasses; he otherwise had a normal retinal exam.

**Table 1:** Laboratory investigation summary of a 14-year-old patient with diagnosis of Prader–Willi syndrome

Laboratory	Result	Reference range
Fasting blood sugar	116 mg/dl	< 100 mg/dl
Luteinizing hormone	< 1 miu/ml	1.1–7 miu/ml
Follicle-stimulating hormone	< 1 miu/ml	1.7–2 miu/ml
Total testosterone	0.75 ng/ml	2.27–10.3 ng/ml
Serum prolactin	10.9 ng/ml	3–25 ng/ml
Thyroid stimulating hormone	3.21 micIU/ml	0.3–5 micIU/ml
Total T3	3.01 nmol/l	1.23–3.07 nmol/l
Total T4	110.7 nmol/l	66–181 nmol/l
Serum 25 (OH) vitamin D	9.7 ng/ml	20–100 ng/ml
Serum cortisol	7.2 µg/dl	3.7–19.4 µg/dl
Adrenocorticotropic hormone	26 pg/ml	10 and 60 pg/ml
24-h urine cortisol	90.23 µg/24 h	28.50–213.70 µg/24 h
High density lipoprotein	32 mg/dl	> 40 mg/dl
Total cholesterol	189 mg/dl	< 200 mg/dl
Low density lipoprotein	137 mg/dl	< 130 mg/dl
Serum triglyceride	98 mg/dl	< 150 mg/dl

Abdominal ultrasound imaging was normal except for nonspecific hepatomegaly of 18 cm with homogenous echo pattern and smooth contour. Brain magnetic resonance imaging (MRI) was unremarkable except for partial empty sella. Genetic testing [SALSA*®* MLPA*®* (Multiplex ligation-dependent probe amplification)] showed an aberrant methylation pattern on 15q11-13 region (PWS region).

The aberrant methylation pattern was seen at the 15q11 region specifically. The study includes assessment of MKRN3, MAGEL2, NDN, SNRPN, UBE3A, ATP10A, GABRB3, and OCA2 genes. It also assessed other genes involved in Prader–Willi-like syndrome including LEPR1, POMC3, SIM1, LEP, SH2B1, MC2R2, MC4R1, MC3R1 and PPARG. There were no significant copy number changes on the genes associated with obesity.

 A timeline graph summarizing developmental and medical history

## Discussion

The diagnosis of PWS is established in a clinically suspected case by identification of abnormal DNA methylation within the Prader–Willi critical region (PWCR) at 15q11.2–q13. Testing should begin with both DNA methylation analysis and oligo-small nucleotide polymorphism (SNP) combination array to establish the diagnosis and identify the molecular cause in most individuals [9].

The management of Prader–Willi syndrome (PWS) symptoms is age dependent and should include both addressing the consequences of PWS and anticipatory advice with a team approach. Management is recommended through consultation with multidisciplinary specialists, and typically starts with neonatologists and is followed by medical geneticists and genetic counselors, primary care physicians, endocrinologists, orthopedists, nutritionists, psychologists, psychiatrists, physical, occupational, and speech therapists, and educators. [10].

Hormone replacement therapy at puberty and beyond includes testosterone, growth hormone, and levothyroxine together with calcium and vitamin D supplementation [6]. Dietary management includes a well-balanced, low-calorie diet, regular exercise, and close supervision to minimize food stealing and prevent obesity. However, lifestyle interventions are often ineffective, particularly in older patients with PWS as well as in patients that have received a late diagnosis [11].

The greater majority of drugs currently available have proven to be ineffective to treat hyperphagia and the resulting excessive weight gain, with the promising exception of glucagon-like peptide-1 (GLP-1) agonists [12]. Successful rapid weight reduction and the use of liraglutide for morbid obesity in an adolescent with Prader–Willi syndrome was reported in 2020 [13]. A systematic review of GLP-1 agonists, which include ten studies on exenatide and liraglutide in patients with Prader–Willi syndrome, showed improvement of body mass index ranging from 1.5 to 16.0 kg/m^2^ [12].

Gastric bypass is not recommended in patients with PWS, as it does not appear to correct the lack of satiety, and complication rates are high. A meta-analysis of 54 articles covering 114 patients with PWS found that bariatric surgery only provides brief relief. Weight loss was greatest in PWS patients 1 year after surgery, but there was no meaningful weight-change percentage after 5 years [14].

Our patient has childhood developmental delay, hypogonadism, small genitalia, short stature, small hands and feet, some facial features of PWS, childhood-onset class III obesity, and vitamin D deficiency. Using consensus clinical diagnostic criteria for PWS 1993, our patient fulfilled six of eight major criteria (excessive weight gain, facial features, hypogonadism, recent learning difficulties, hyperphagia, and the aberrant methylation in the PWS region) and five of eleven criteria (characteristic behavior problems, short stature, small hands, eye refractory error, and speech articulation). Summing up these criteria created a score for the patient of 8.5, which is enough for diagnosis [15]. Nevertheless, 17% of 90 patients with a molecular PWS diagnosis did not meet the 1993 consensus clinical diagnostic criteria in one study. Other suggested new clinical criteria to prompt DNA testing for PWS include presence of cognitive impairment, excessive eating, central obesity, and hypothalamic hypogonadism [1]. In addition to the clinical features of PWS, our patient had an aberrant methylation pattern seen at PWS region on genetic analysis, which confirms the diagnosis.

He is being treated with strict lifestyle intervention (advice on meal and regular exercise), parenteral testosterone treatment, a high dose of vitamin D, and spectacles, as well as endocrinologist, dietician, ophthalmologist, and psychiatrist follow-up. Growth hormone and GLP-1 agonists are not locally available. He followed up at our hospital for total of 4 months; his last visit was on 28 August 2023. He had subjective improvement of well-being, and his serum vitamin D and glycemia normalized. However, no improvement of weight loss was achieved so far. With regard to rare possible recurrence, his mother is already at menopause and all his siblings are healthy. We are following him for additional possible comorbidities, which include obstructive sleep apnea, osteoporosis, seizure, and decreased saliva production. Further medical and surgical management options are being discussed with the caregivers.

## Conclusion

Prader–Willi syndrome should be considered in a child who presents with progressive weight gain and other typical clinical features, such as cognitive impairment, excessive insatiable eating, or hypothalamic hypogonadism, to diagnose it as early as possible. Early lifestyle intervention is paramount to decrease morbid obesity and associated comorbidities. After a large amount of weight gain is achieved, antiobesity medications and bariatric surgery do not produce satisfactory weight loss outcomes.

## Data Availability

All data sets on which the conclusions of the case report are based are to be available as a medical record document and available from the corresponding author on reasonable request from the editors.

## Abbreviations

PWS: Prader–Willi syndrome
GLP: Glucagon-like peptide
DNA: Deoxyribonucleic acid
MRI: Magnetic resonance imaging
T3: Triiodothyronine
